# Syphilis in the Ninth Century

**Published:** 1883-07

**Authors:** 


### SYPHILIS IN THE NINTH CENTURY.

Between the years A. D. 806 and 810, an Emperor of Japan commanded his court physicians, Abemanus and Idzumo Kirosada, to collect in one volume all extant records of native medicine and surgery. (British Medical Journal.) A manuscript copy of this work, for centuries forgotten, although the facts of its origin were recorded in Japanese history, was found in 1827, by a priest, in a provincial Buddhist temple. Dr. Scheube, of Leipzig, has recently examined this work, and, in an article published in a recent number of Virchow's Archiv (Louisville Medical News) has shown its undoubted authenticity and its high value from a scientific point of view. It was written long before Chinese ideas had penetrated into Japan and influenced native practitioners. The most interesting passages are descriptions of local and general affections, which clearly prove that syphilis and several allied disorders were well known to the ancient Japanese. Chancroid and phagedenic chancre are clearly described, as well as a "swelling on the penis, the size of a millet-seed," followed by eruptions, feverishness, pains in the bones and head, blindness, swelling of the testicles, and other very familiar symptoms. These were observed to continue for many years. The passages of this work, called the Daidorui Thiu-ho, which relates to the treatment of these symptoms, have not yet been translated into English. Herbs alone appear to have been used, and without much success ; mercurial treatment was introduced at a comparatively recent date from Europe. The ancient Japanese surgeons do not appear to have recognized the venereal origin of the disease they describe, although the Daidorui distinctly traces all the secondary symptoms to "the poison from the affected organ."— *Weekly Medical Review.*

